# Colchicine and Cardiovascular Outcomes: a Critical Appraisal of Recent Studies

**DOI:** 10.1007/s11883-021-00932-5

**Published:** 2021-05-10

**Authors:** Maciej Banach, Peter E. Penson

**Affiliations:** 1 Department of Hypertension, Medical University of Lodz (MUL), Rzgowska 281/289, 93-338 Lodz, Poland; 2 Polish Mother’s Memorial Hospital Research Institute (PMMHRI), Lodz, Poland; 3 Cardiovascular Research Centre, University of Zielona Gora, Zielona Gora, Poland; 4 School of Pharmacy and Biomolecular Sciences, Liverpool John Moores University, Liverpool, UK; 5 Liverpool Centre For Cardiovascular Science, Liverpool, UK

**Keywords:** Colchicine, Randomised controlled trials, Acute coronary syndromes, Chronic coronary syndromes

## Abstract

**Purpose of Review:**

Recent studies have demonstrated an important role for inflammation in the pathogenesis of atherosclerotic cardiovascular disease. Several studies have investigated the efficacy of colchicine (a widely used and safe anti-inflammatory drug) in patients with atherosclerosis. This review explains the rationale for the use of colchicine in this setting and critically appraises recent outcome trials.

**Recent Findings:**

Two large randomised-controlled trials LoDoCo2 (included patients with chronic coronary syndromes) and COLCOT (acute coronary syndromes) have demonstrated reductions in atherosclerotic cardiovascular events, but not mortality. A smaller study (COPS) found no beneficial effect of colchicine but was probably underpowered.

**Summary:**

Colchicine is effective at reducing cardiovascular events in chronic and acute coronary syndromes, although reductions in all-cause mortality have not been demonstrated during the period of follow-up in trials to date. Mild gastrointestinal symptoms are the most commonly reported adverse effects, although in well-designed randomised controlled trials these are relatively uncommon.

## Introduction

Colchicine, an alkaloid derived from *Colchicum autumnale* (autumn crocus) [1], is an extremely old drug with a remarkable capacity for reinvention. Preparations of crocus have been used to treat joint pain for over 3500 years, with the active ingredient being discovered in the 1800s. Today, colchicine is used to treat gout, familial Mediterranean fever and pericarditis [2] and has been investigated for use in the management of coronavirus 2019 (COVID-19) [3] with promising initial results recently reported from the investigators of the ColCORONA study [4, 5]. To add to this ostensibly eclectic list, studies have investigated the potential for the use of colchicine in the prevention of atherosclerotic cardiovascular disease [6–14, 15••, 16, 17••, 18••], the subject of this review.

Inflammation unites the pathology of conditions for which colchicine has established or putative indications. The mechanism of action of colchicine is complex and beyond the scope of this review. However, colchicine is a microtubule inhibitor and potentially inhibits the NLRP3 inflammasome at various points [2, 19] and has been shown to reduce pro-inflammatory cytokines including interleukin-1β (IL-1β), interleukin-18 (IL-18) and interleukin-6 (IL-6), as well as the inflammatory marker C-reactive protein (CRP) in a variety of experimental models and clinical settings [2, 19]. Recent findings relating to atherosclerosis biology and epidemiological research in cardiovascular disease strongly suggest that such effects are likely to be beneficial in the prevention of atherosclerotic cardiovascular disease in susceptible individuals. The rationale for this approach is discussed below, followed by a summary and critical overview or recent clinical research.

## Inflammation and Cardiovascular Disease

Since the mid-twentieth century, remarkable progress has been made in the prevention of cardiovascular disease through the identification and management of modifiable risk factors. Observations by Gofman [20] and the Framingham investigators [21] relating to the association between elevated low-density lipoprotein cholesterol (LDL-C) and atherosclerotic cardiovascular disease, combined with improved understanding of the role of LDL-C deposition in arterial walls in atherosclerosis, led first to dietary approaches to LDL-C reduction, by reducing dietary saturated fat, and later to the development of effective lipid-lowering drugs. Statins, inhibitors of endogenous LDL-C production, reduce the frequency of atherosclerotic cardiovascular events by approximately 25% for each mmol/L reduction in LDL cholesterol, for each year of therapy [22]. Monoclonal antibody inhibitors of proprotein convertase subtilisin/kexin type 9 (PCSK9) upregulate hepatic LDL receptors, increasing clearance of LDL from the plasma, causing a substantially greater reduction in LDL-C than statins and further reductions in cardiovascular events [23]. The causal relationship between LDL-C and atherosclerosis has been firmly established [24], and prevention of cardiovascular disease should be aimed towards reducing lifelong exposure to LDL-C, according to the principle ‘*Lower is better for longer*’ [25, 26] and the new concept of ‘*the earlier the better*’ [26–28].

Nevertheless, even optimal management of LDL-C does not eliminate the risk of atherosclerotic cardiovascular events, and recent attention has turned towards the identification and management of the ‘residual’ risk. The most promising approach to date has been the targeting of inflammation. The suggestion that inflammation is an important component of atherosclerotic disease is not new. Indeed, Virchow made this observation from a pathophysiological perspective in the nineteenth century [29], an observation that has been repeated and extended by others, particularly Ross, Libby and Ridker [30, 31], and which is supported by epidemiological evidence demonstrating the association between elevated CRP and atherosclerotic events, particularly in individuals with low LDL-C [32••]. However, only recently have these observations been exploited to reduce cardiovascular risk. This delay can be explained by previous lack of knowledge of the precise molecular mechanisms involved in the inflammatory processes in atherosclerosis, lack of selective inhibitors and scepticism of this approach arising from poor outcomes when non-specific anti-inflammatory agents such as corticosteroids have been used in the setting of acute myocardial infarction [33], and increased incidence of cardiovascular events in individuals treated with anti-inflammatory inhibitors of cyclooxygenase 2 (Cox-2) [34].

Proof of concept of the ‘inflammatory hypothesis’ of atherosclerosis has come in particular from the Justification for the Use of Statins in Primary Prevention: an Intervention Trial Evaluating Rosuvastatin (JUPITER) and Canakinumab Anti-Inflammatory Thrombosis Outcomes Study (CANTOS) studies, leading to the understanding that atherosclerosis is a lipid-driven inflammatory condition [35]. In JUPITER, 17,802 heathy individuals with what was then considered to be ‘normal’ LDL-C (< 130 mg/dl, <3.4 mmol/l), but elevated CRP, were randomised to receive rosuvastatin or placebo. Rosuvastatin reduced CRP, LDL-C and cardiovascular events. These results are hard to interpret, especially in light of the recent findings of the benefits of ever-lower LDL-C; however, the results of JUPITER sparked interest in the anti-inflammatory mechanisms of statins [36, 37] and the role of inflammation in the pathophysiology of atherosclerosis. The CANTOS study evaluated the efficacy of canakinumab, a monoclonal antibody inhibitor of IL-1β (and like colchicine, an existing treatment for gout) in 10,061 patients with previous myocardial infarction and elevated CRP. As expected, canakinumab reduced inflammatory markers but had not effect on lipid profiles. Canakinumab treatment caused a small but statistically significant reduction in cardiovascular events and thus demonstrated that anti-inflammatory agents and targeting of the NLRP3 inflammasome [38, 39] could reduce the risk of atherosclerotic cardiovascular disease (ASCVD) events [40]. The relatively small benefit of canakinumab and its high acquisition cost resulted in a decision not to pursue regulatory approval for the use of canakinumab for cardiovascular indications; however, CANTOS opened the door to the use of other drugs acting on the same or related targets. In particular, substantial effort has been directed towards repurposing existing drugs, thereby benefiting from the established safety profile and (where drugs are available generically) low acquisition costs. In this context, colchicine was identified as a promising therapeutic candidate: it is cheap and has a long-established safety record.

In the treatment of gout, colchicine acts by inhibiting the components of the NLRP3 inflammasome, which is activated in response to the deposition of sodium urate crystals in soft tissue [41]. Similarly, atherosclerosis is characterised by NLRP3 activation following deposition of cholesterol crystals in the walls of blood vessels. The NLRP3 pathway is complex and involves the activation of IL-6 by parallel mechanisms involving IL-18 and IL-1β. Canakinumab targets IL-1β, and recent findings have demonstrated that residual risk in canakinumab-treated patients is driven by IL-18-mediated elevation of IL-6 [42]. In this context, the demonstrated reduction of IL-18, IL-1β and IL-6 by colchicine make it a very attractive theoretical candidate for the reduction of inflammatory risk in atherosclerosis.

## Clinical Studies of Colchicine in ASCVD

A variety of studies have been conducted with the use of low-dose colchicine (typically 0.5–1 mg/day) in a range of ASCVD patients (Table 1). Most studies have been of relatively short duration and have focused on surrogate outcomes in relatively small populations. The results from these studies have been somewhat inconsistent, probably reflecting differences in sample size and study design and heterogeneity in patient populations. Whilst some studies have shown no effect of colchicine on inflammatory markers [6, 11, 13] or flow-mediated dilation [12], other studies have demonstrated reductions in IL-6, IL-18, IL-1β, caspase-1 and increased lumen diameter [8–10, 14]. However, it is not possible to draw inferences from these trials to inform patient care, both because of the methodological limitations listed above and because of the difficulty in translating inflammatory markers to cardiovascular outcomes or predicting outcomes based upon expected pharmacological effect. This problem is exemplified by the Cardiovascular Inflammation Reduction Trial (CIRT) in which treatment with methotrexate, an alternative anti-inflammatory drug, had no effect on inflammatory markers or atherosclerotic events [46]. Previous observational studies in individuals with rheumatoid arthritis had suggested that methotrexate was associated with improved cardiovascular outcomes, compared with other anti-rheumatic drugs. However, the interpretation of such studies is inevitably complicated by confounding by indication and unmeasured biases [47]. However, it has been suggested that the prevention of atherosclerotic events by methotrexate may be limited to this population with extensive inflammation, hence the ineffectiveness of the drug in the comparatively low-risk population in CIRT [47].

**Table 1:** Summary of characteristics and findings of randomised controlled trials and meta-analyses of colchicine in atherosclerotic cardiovascular disease

Author	Year	Study name	Ref	Population	Number of subjects colchicine/control	Colchicine dose	Duration of follow-up	Colchicine efficacy	Colchicine safety
Randomised controlled trials									
Raju	2012	NA	[6]	ACS	40/40	1 mg/day	1 month	No effect on CRP	↑diarrhoea
Nidorf	2013	LoDoCo	[7]	CAD	282/250	0.5 mg/day	36 months	↓ CV events	↑GI AEs
Deftereos	2013	NA	[8]	CAD-PCI + DM	100/96	0.5 mg twice daily	1 month	↓ restenosis ↑lumen diameter	↑GI AEs
Martinez	2015	NA	[9]	CAD	34/39	1 mg + 0.5 mg	1 day	↓ IL-1ß, IL-18 and IL-6	NR
Robertson	2016	NA	[10]	ACS	10/11	1 mg + 0.5 mg	2 days	↓ IL-1ß and caspase-1	NR
Akodad	2017	COLIN	[11]	STEMI-PCI	23/21	0.5 mg twice daily	1 month	No effect on CRP	↑GI AEs
Kajikawa	2019	NA	[12]	CAD	14/14	0.5 mg/day	14 days	No effect on FMD	NR
Hennessy	2019	NA	[13]	AMI	119/118	0.5 mg/day	1 month	No effect on CRP or IL-6	↑GI AEs
Shah	2020	COLCHICINE-PCI	[14]	CAD-PCI	206/194	1.2 mg stat + 0.6 mg/day	1 month	↓ IL-6 and CRP	↑GI AEs
Tardif Bouabdallaoui	2019 2020	COLCOT	[15••, 16]	Post-MI	2322/2339	0.5 mg/day	22.7 months	↓MACE	↑Pneumonia ↑ GI AEs
Nidorf	2020	LoDoCo2	[17••]	CAD	2762/2760	0.5 mg/day	28.6 months	↓MACE	Well tolerated
Tong	2020	COPS	[18••]	ACS	396/399	0.5 mg twice daily for 1 month then 0.5 mg /day	12 months	No effect on MACE	↑ GI AEs
Meta-analyses									
Al-Abdouh	2020	NA	[43]	CAD	3096/3058	0.5–1 mg daily	1 month–3 years	No effect on MACE	NR
Samuel	2020	NA	[44]	CAD	5774/5820	0.5 daily–twice daily	1 month–3 years	↓MACE	Well tolerated
COVID-19									
	2021	ColCORONA	[45]	COVID-19	2235/2253	1 mg/day	30 days	↓Composite of death and hospitalisation	↑ GI AEs

*ACS* acute coronary syndromes, *AE* adverse effect, *CAD* coronary artery disease, *CRP* C-reactive protein, *FMD* flow-mediated dilation, *GI* gastrointestinal, *IL* interleukin, *MACE* major adverse cardiovascular events, *NA* not applicable, *NR* not reported

The first outcome trial of colchicine in ASCVD was the Low Dose Colchicine for Secondary Prevention of Cardiovascular Disease (LoDoCo) study [7], a remarkably prescient investigation, published in 2013, before CANTOS had definitively demonstrated the causal relationship between IL-1β and adverse cardiovascular events. In a prospective, randomised study, 532 patients with stable coronary artery disease, the majority of whom were treated with statins and antiplatelet drugs, were randomised to colchicine treatment (0.5 mg/day) or control and were followed for a median of 3 years for a composite primary endpoint of acute coronary syndrome, out-of-hospital cardiac arrest or non-cardioembolic ischemic stroke. The endpoint occurred in 5.3% of participants in the colchicine group, and 16.0% of participants assigned to control (hazard ratio (HR): 0.33; 95% confidence interval (CI) 0.18 to 0.59; *p* < 0.001). Unfortunately, the study had several weaknesses which limit the extent to which the trial could influence clinical practice. The small sample size reduces the external validity of the data and increases the likelihood of drawing erroneous findings due to chance. Importantly, bias may have been introduced by the absence of placebo control or blinding of patients to their treatment allocation. Finally, the relatively short duration of follow-up does not permit conclusions to be drawn with respect to the long-term safety or efficacy of this therapeutic strategy.

The subsequent LoDoCo2 randomised-controlled double blind trial was substantially larger and more rigorously conducted. The LoDoCo2 investigators randomised 5522 patients with chronic coronary disease (evidence of coronary disease from angiography or coronary-artery calcium scan, and a stable clinical condition for at least 6 months) to receive 0.5 mg colchicine daily or placebo [17••]. Patients were followed up for a median of 28.6 months for a composite endpoint comprising cardiovascular death, myocardial infarction (MI), ischaemic stroke or coronary revascularization. Colchicine treatment caused a reduction in the composite endpoint (HR, 0.69; 95%CI, 0.57–0.83, *p* < 0.001). The Kaplan-Meier curves show a clear divergence between the colchicine and placebo groups very early in the trial (circa 6 months), with the gap increasing with time [17••] . Nevertheless, the duration of follow-up was relatively short, and the data cannot be extrapolated beyond the trial duration.

In both colchicine and placebo groups, revascularisation procedures accounted for the biggest proportion of the composite endpoint [17••]. Unlike objectively adjudicated cardiovascular death, MI or stroke (‘hard’ endpoints), revascularisation is somewhat ‘soft’ because the decision to undertake revascularisation is made by a doctor and relies upon patient consent. However, as the patients and treating physicians were unaware of treatment allocation, there is no particular reason to expect that this weakness would bias the results in any particular direction. Safety aspects are discussed in a later section.

The generalisability of the results of LoDoCo2 is limited by the small proportion of female subjects (15%) enrolled in the trial. Given the complex pharmacological profile of colchicine, it would have been interesting from a scientific perspective to investigate the time course of key biomarkers throughout the trial to determine whether the drug is likely to be exerting its effects through the expected mechanisms. Unfortunately, such analysis was not possible as measurements of inflammatory markers, blood pressure and lipids were not made at baseline [17••].

In contrast to the LoCoCo2 trial, which recruited individuals with chronic coronary disease, the COLCOT trial recruited 4745 individuals who had experienced an acute coronary syndrome within 30 days, had undergone any planned revascularisation procedures and were treated according to national guidelines, which included the recommendation for high-intensity statin therapy [15••]. The composite endpoint comprised cardiovascular death, resuscitated cardiac arrest, MI, stroke or revascularisation. Treatment allocation was randomised, and neither participants nor treating physicians were aware of treatment allocations. The median duration of follow-up was slightly shorter than LoDoCo2 (22.6 v. 28.6 months). Incidence of the primary endpoint was lower in the treatment group (HR, 0.77, 95% VI, 0.61–0.96, *p* = 0.02). The proportion of women in the COLCOT trial was also small (19%), and as with LoDoCo2, incomplete monitoring of biomarkers precludes mechanistic investigation of the observed effects of colchicine on outcomes.

Another recent randomised-controlled, double blind study, the Australian Colchicine in Patients with Acute Coronary Syndromes (COPS) clinical trial has also investigated the efficacy of colchicine post ACS. The authors randomised 795 patients, across 17 centres, to receive colchicine (0.5 mg twice daily for 1 month, then 0.5 mg daily for 11 months) or placebo [18••]. The primary outcome was a composite of all-cause mortality, ACS, urgent revascularisation and stroke. The authors found no statistically significant benefit of treatment on the primary outcome at 12 months of follow-up. This result is clearly in contrast to COLCOT, which demonstrated a beneficial effect of colchicine on a similar primary endpoint, with a similar patient population, and LoDoCo2 which recruited patients with chronic coronary syndromes. The most likely explanation for the difference is the low statistical power in the COPS trial, which was an investigator-led trial, and despite setting up 17 recruitment sites, budgetary issues limited the ability of the investigators to recruit and follow up patients [18••]. As a result, the analyses in COPS were based upon 62 primary endpoint events, compared to a combined total of 752 events in COLCOT and LoDoCo2 [48]. Therefore, the results of this trial also should be treated with caution and would be most useful when synthesised with the findings from related studies in a meta-analysis, although the higher initial dose in COPS may result in substantial heterogeneity and may explain differences between the findings of the three trials.

None of the randomised-controlled outcome trials (LoDoCo2, COLCOT and COPS) demonstrated a reduction in mortality associated with colchicine treatment. It is not clear whether this reflects the fact that colchicine is truly neutral with respect to mortality or whether it is explained by insufficient statistical power. In the COPS study, colchicine treatment was, in fact, associated with an increase in mortality compared to placebo (8 v. 1 deaths). Similarly, in LoDoCo2, there was a numerically greater number of deaths in the treatment group (73 v. 60 deaths; HR, 1.21; 95%CI 0.86–1.71). In COLCOT, the number of deaths was almost identical (43 v. 44 deaths; HR, 0.98; 95%CI 0.64–1.49). The wide confidence intervals around these estimates indicate that it is likely that any differences are due to the play of chance. Given the long history of colchicine use, the numerical increases in mortality on treatment in two trials should not be a particular concern, but mortality should be followed up, where possible, in existing cohorts to gather more data [15••, 17••, 18••, 48].

These findings have been confirmed by a recently completed study-level meta-analysis, conducted on behalf of the International Lipid Expert Panel, which is currently in peer review. The meta-analysis included 12 randomised-controlled trials, with a total of 12,989 patients, with a mean follow-up of 22.6 months. Colchicine treatment was associated with a lower risk of major adverse cardiovascular events (RR 0.67; 95%CI 0.6–0.92, *P* = 0.004); CRP and IL-6 (but not other inflammatory markers) were significantly reduced. There was no apparent effect of colchicine upon mortality [49].

Each of the randomised-controlled outcome trials (LoDoCo2, COLCOT and COPS) also measured safety outcomes. As would be expected with a drug which acts by reducing inflammatory/immune mechanisms, particular attention was paid to the possibility of increased rates of infection in colchicine-treated patients. In COLCOT, treatment was associated with a small increase in the incidence of pneumonia (0.9% v. 0.4%), whereas this effect was not observed in LoDoCo2 [15••, 17••, 18••, 48].

Almost all trials reported increased frequency of gastrointestinal adverse effects (particularly diarrhoea) in colchicine-treated patients. As this effect has been consistently demonstrated in randomised studies, it is likely to indicate a causal relationship. However, it is important not to overestimate the potential importance of this effect to limit treatment. In the LoDoCo2 trial, a pre-randomisation run-in period was used, in which potential participants who experienced adverse effects left the trial before allocation to colchicine or placebo [17••]. Thus, the reported adverse effects in the randomised portion of the trial are likely to underestimate the expected prevalence in clinical practice. However, the COLCOT trial used no such run-in and therefore perhaps gives a better estimate. In this trial, diarrhoea was reported in 9.7% of patients in the treatment group compared with 8.9% in the placebo group. Thus, this is a small effect, albeit a well-known one. Extensive evidence from statin trials has indicated that the *drucebo* effect (whereby a patient experiences adverse drug reactions as a result of expecting such effects) accounts for a substantial proportion of reported muscle pain in patients receiving these drugs for hypercholesterolemia. This can limit the adherence to life-saving preventative drugs [50–52]. Because gastrointestinal symptoms are common (so symptoms from unrelated causes are misattributed to colchicine) and can be triggered psychosomatically, it seems likely that the drucebo effect might be expected to apply to colchicine therapy. Thus, if colchicine is to be used widely in the prevention of ASCVT, it is important to make patients aware of the relative infrequency and reversibility of adverse effects.

All the three outcomes trials to date have been conducted in ‘high-risk’ populations [15••, 17••, 18••]. It would be interesting in the future to determine whether colchicine reduces the incidence of ACVD events in a lower-risk primary-prevention population. Whilst such an approach has potential benefits, it also presents several challenges. The absolute benefit of any intervention is likely to be smaller in a low-risk population, so the potential for adverse effects might weigh more heavily against treatment in a low-risk population. Furthermore, as colchicine is no longer covered by patents, a new indication is unlikely to generate substantial revenue; thus, funding for clinical trials may be hard to secure. However, the evaluation of colchicine in larger populations could be achieved by including combining it with another intervention in a randomised trial with factorial design. Alternatively, the low cost of colchicine could make it suitable for evaluation together with other anti-atherosclerotic agents in a polypill preparation [53].

## Conclusions

Low-dose colchicine has been shown to reduce cardiovascular events in patients with acute and chronic coronary syndromes in two large outcomes trials, in which the drug was generally well tolerated. No indication has been found that colchicine reduces mortality, although additional studies or longer follow-up of existing trials is required to possibly confirm this fact. In smaller trials, the effects of colchicine have been variable with respect to the effect of the drug on outcomes and inflammatory markers.

## Abbreviations

ASCVD: Atherosclerotic cardiovascular disease
CANTOS: Canakinumab Anti-Inflammatory Thrombosis Outcomes Study
CI: Confidence interval
CIRT: Cardiovascular Inflammation Reduction Trial
COLCOT: Colchicine Cardiovascular Outcomes Trial
ColCorona: Colchicine Coronavirus SARS-CoV2 Trial
COPS: Colchicine in Patients with Acute Coronary Syndromes
COVID-19: Coronavirus 2019
Cox-2: Cyclooxygenase 2
CRP: C-reactive protein
HR: Hazard ratio
IL-1β: Interleukin-1β
IL-6: Interleukin-6
IL-18: Interleukin-18
JUPITER: Justification for the Use of Statins in Primary Prevention: an Intervention Trial Evaluating Rosuvastatin
LDL: Low-density lipoprotein
LDL-C: Low-density lipoprotein cholesterol
LoDoCo: Low Dose Colchicine for Secondary Prevention of Cardiovascular Disease
MI: Myocardial infarction
PCSK9: Proprotein convertase subtilisin/kexin type 9
